# Variation in the lexical semantics of property concept roots: Evidence from Wá⋅šiw

**DOI:** 10.1007/s11049-025-09671-7

**Published:** 2025-07-08

**Authors:** Emily A. Hanink, Andrew Koontz-Garboden

**Affiliations:** 1 Linguistics, Indiana University, S. Indiana Ave, Bloomington, 47405 IN USA; 2 Linguistics and English Language, University of Manchester, Oxford Rd, Manchester, M13 9PL UK

**Keywords:** Lexical semantics, Roots, Property concepts, Categorization, Possessive predication

## Abstract

Whether the lexical semantics of property concepts (words canonically expressed as adjectives in languages with that category; Dixon 1982, Thompson 1989) show variation is a matter of recent debate. At one end of the analytical spectrum, Francez & Koontz-Garboden (2017) contend that their meanings may vary in a way revealed by superficial morphosyntactic behavior. At the other end, Menon & Pancheva (2014) argue that they are universally built on abstract mass-denoting roots, a commonality that can be obscured by (covert) morphosyntax introducing possessive meaning. On the basis of differing strategies for property concept verb formation in Wá⋅šiw (isolate/Hokan, USA), we argue in this paper that there is evidence for variation in the lexical semantics of property concept roots, with some denoting predicates of individuals and others having abstract mass-type meanings, contrary to universalist assumptions. Crucially, the behavior of property concept verb formation in Wá⋅šiw lends itself to an analysis in which possessive semantics is implicated only when it is morphologically observable. By drawing an analogy to canonical possession in the language, we argue moreover that this extra morphology in property concept verbs is best understood as a light verb that both directly categorizes property concept roots and introduces a possessive semantics. These observations provide evidence for the claim that at least some variation in this domain is underpinned by variation in lexical semantics, and more generally for the idea that variation in the lexical semantics of open-class elements drives at least some variation in morphosyntax.

## Introduction

Thompson (1989) coined the term *property concepts* for the set of lexical items (words and bound roots) across languages that translate English adjectives, even when instantiated by other categories—a common eventuality first documented in crosslinguistic context by Dixon (1982) and discussed by many since (Thompson 1989; Hengeveld 1992; Croft 1991, 2001; Bhat 1994; Wetzer 1996; Stassen 1997; Beck 2002; Baker 2003).^1^ More recently, there has been examination of variation in the lexical semantics of property concepts, with the question whether variation in meaning and category are at all linked to one another, either across or within languages (Menon and Pancheva 2014; Francez and Koontz-Garboden 2015, 2017).

In particular, Koontz-Garboden and Francez (2010) and Francez and Koontz-Garboden (2015, 2017) argue for two different kinds of meanings for property concepts crosslinguistically, with these meanings partially restricted by syntactic category. One type of meaning, familiar from adjectives in English and other well-studied languages, characterizes sets of ordinary individuals. That this is the case is seen straightforwardly by the fact that they can serve as ordinary (nonverbal) predicates, as in (1), which is true just in case Kim is in the (contextually restricted) set of strong individuals:

1. Kim is strong.

Others, which are commonly categorized as nouns (but also bound roots, as in Ulwa; Koontz-Garboden and Francez 2010; Francez and Koontz-Garboden 2015), instead have an abstract mass-type meaning. Koontz-Garboden and Francez argue that such a meaning is diagnosed by a requirement for possessive morphosyntax in predication, as a possessive semantics is necessary to generate a meaning that can be attributed to an ordinary individual (the subject). The Hausa (Chadic) in (2) exemplifies a construction along these lines, in which the nominal property concept (*ƙarfī*, ‘strength’) is possessed via the preposition  ‘with’ before being predicated of the subject. (3) shows that the same strategy is used to express canonical possession of an ordinary entity.

(2)
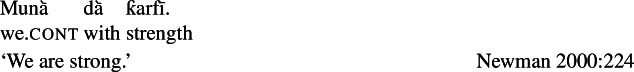

(3)
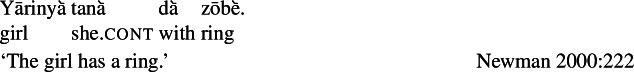

The idea that such nouns do not have the same kinds of meanings that their adjectival counterparts do can be confirmed by the consideration of minimal pairs in ordinary predicative contexts, such as (4), where (4a,b) do not have the same truth conditions. In particular, (4b) has the meaning not that Kim is hungry, but rather that Kim is hunger (whatever that means). Compare (5), which has truth conditions more on a par with (4a).

(4)
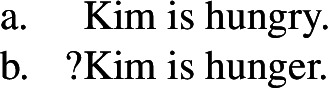

(5) Kim has hunger.

According to Francez and Koontz-Garboden, it is a point of variation whether property concepts characterize sets of individuals or have an abstract mass-type meaning. Building on this work, Menon and Pancheva (2014) adopt their analysis of abstract mass-denoting property concept nouns, but argue for a much stronger, universal claim: *All* property concept roots begin life as such mass-denoting expressions, and must be overtly or covertly possessed in order to predicate of individuals. That is, while Francez and Koontz-Garboden (2017) contend that property concepts can vary in their meaning, Menon and Pancheva (2014) argue that all property concept words are derived from roots with the same kind of meaning prior to being possessed, with variation in the morphology and syntax of predication with them tied to the inventory and phonological realization of functional heads. These perform a variety of roles including introducing possession, thereby turning abstract mass roots into predicates of individuals whilst categorizing them as nouns, verbs or adjectives. Alternatively, some such categorizers fail to introduce possessive meaning, in which case possession is introduced in other ways (e.g., with a *have* verb or similar). At play, then, is the locus of variation: is it tied exclusively to variation in functional vocabulary? Or is there variation in this domain at the level of the lexical semantics of open-class items, i.e., property concepts themselves?

Recently, Hanink et al. (2019) and Hanink and Koontz-Garboden (2024) show that there is evidence that at least *some* such variation is a consequence of variation in functional vocabulary, thereby raising the question whether *all* of it might be, as Menon & Pancheva conjecture. In this paper, we show that this cannot be the case. In particular, we show that there is evidence for at least some variation in property concept root meanings, with some denoting predicates of individuals and others with abstract mass-type meanings, as originally envisaged by Francez and Koontz-Garboden. We argue for this state of affairs on the basis of data from property concept verbs in Wá⋅šiw (also spelled Washo(e); isolate/Hokan, USA).

In Wá⋅šiw, property concepts are always verbal, but come in three different shapes ((6)–(8)) that differ in morphosyntactic complexity:^2^

(6)
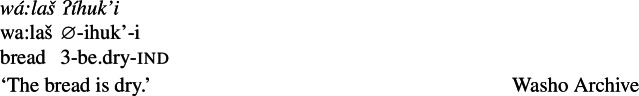

(7)
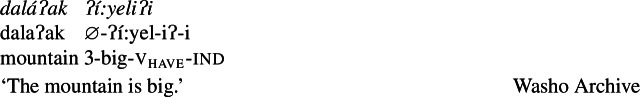

(8)
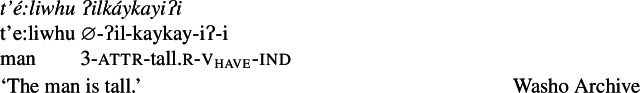

In what follows, we argue that the above variation in morphology implicates possession only in cases where it is overtly marked by the suffix *-iʔ* (as in (7) and (8)). Drawing on core insights from Francez and Koontz-Garboden, we argue that the role of this suffix in property concept word formation is to introduce a possessive relation in which the possessum is an otherwise bound property concept root. In cases where *-iʔ* is absent (6), this is because the root is born of the right type to predicate of individuals, obviating the need for possession. Furthermore, we show that attributing an abstract mass-type meaning to roots that do not take this suffix leads to incorrect predictions. It follows from this that two kinds of root meaning are required—the mass-type and the directly predicative type. On this basis, we argue against the universalist view put forward by Menon and Pancheva (2014) according to which all property concept roots are mass-denoting, with possession being required even in cases where it is not overt.

The structure of this paper is as follows. In Sect. 2, we lay out relevant background contrasting previous views on the nature of property concept root meaning. In Sect. 3 we present the empirical overview of property concept word formation in Wá⋅šiw. In Sect. 4 we present our analysis of two possible property concept root types in the language: those that denote relations between individuals and states, and those that denote sets of states alone, of which only the latter require possessive morphology in order to act as predicates of individuals. In Sect. 5 we extend our analysis to the most complex type of property concept formation in the language (8), offering evidence to support our view from the behavior of property concept roots in resultative bipartite verbs (Jacobsen 1980). In Sect. 6 we make more explicit our morphosyntactic analysis of possessive predication in the language and develop an analysis in which *-iʔ* is a functional, categorizing light verb v_have_. Section 7 concludes and situates our findings within a broader theoretical context.

## Background and basic proposal

In this section we offer an overview of previous literature relating possession to the attribution of the meanings underlying property concepts to ordinary individuals, namely the proposals put forward by Koontz-Garboden and Francez (2010), Francez and Koontz-Garboden (2015, 2017), and Menon and Pancheva (2014). These approaches can be delimited to a certain extent by the point at which possession is introduced: at the sentential level or the word level. Koontz-Garboden and Francez (2010) and Francez and Koontz-Garboden (2017) focus mostly on sentential morphosyntax, while Menon and Pancheva (2014) substantially extend the possession analysis in the context of categorization.

### Possession at the sentential level

Francez and Koontz-Garboden (2015, 2017) argue that it is a point of semantic variation that property concepts can be lexicalized either as predicates of ordinary individuals or as *qualities*—abstract mass-type meanings. Adjectives in familiar languages such as English are an example of the former, though analytic details vary according to the particular theory of adjectival meaning, for example, those that are degreeless (9a)—invoking evaluation within a context (c)—vs. degree-based (9b)—invoking comparison to some salient degree standard (s_*G*_) (see Beck et al. 2010; Bochnak 2015):

(9)
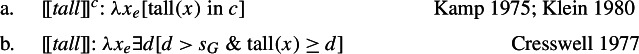

Because of their semantic type, such predicates (in either approach) can be related to individuals by canonical predication with a copula, for example *be* in English and  in Hausa:

(10) Kim is tall.

(11)
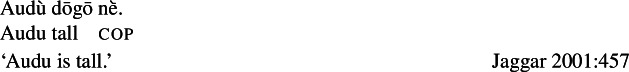

*Qualities* on the other hand, such as the meanings of *hunger* and *strength* in English (and most property concepts in Hausa such as (2), along with others discussed by Francez and Koontz-Garboden), do not characterize ordinary individuals, but rather abstract notions. Though Francez and Koontz-Garboden model qualities with *portions* in the spirit of Link’s (1983) treatment of mass nouns, they are naturally treated as predicates of states (Baglini 2015; Wellwood 2015, 2019; Bochnak et al. 2020; Zato 2020) if states have all the order-theoretic properties that Francez and Koontz-Garboden assume for portions, as for example Bochnak et al. (2020) assume. Crucial among these are a part/whole ordering, from which their mass-type properties follow, and a size-ordering, from which their gradability follows.^3^ On this view, a noun such as *hunger* denotes a set of (part/whole, and size-ordered) states and has the meaning in (12):

(12)
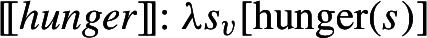

Because such nouns characterize states, rather than participants in them, such nouns are necessarily predicated with a possessive morphosyntax crosslinguistically. Francez and Koontz-Garboden (2017) identify several strategies that languages employ to encode possession of qualities. Their general observation is that any strategy a language has for possessive predication can also be found with possession of nominal property concept words with a quality-type meaning.^4^ For example, beyond the prepositional possession strategy already shown in the case of Hausa above ((2) and (3)), other languages encode possession in the verb, as in Spanish (13a), or by means of an existential construction, as in Bisa (Mande) (14)[a]. The same constructions are found with possession of property concept nouns, as illustrated by (13b) and (14)[b], respectively.

(13)

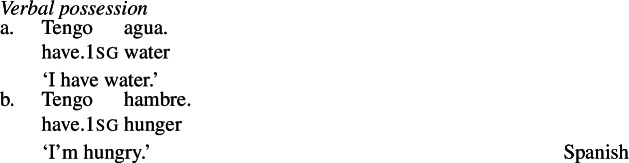

(14)

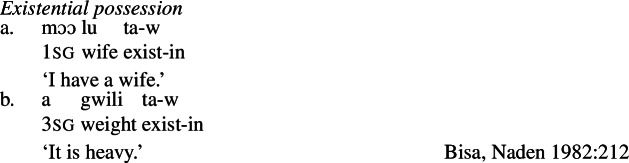

Nouns such as Spanish *hambre* ‘hunger’ and Bisa *gwili* ‘weight’ cannot be predicated of ordinary individuals, because they denote sets of states, rather than of ordinary individuals. To predicate one of these of an ordinary individual will therefore give rise to the meaning that the individual *is* a Davidsonian state. Such is the reason, Francez and Koontz-Garboden claim, that a sentence such as *Kim is hunger* is odd. This is where possession comes into play—it relates the individual to the state in a way that gives rise to the truth conditions found with predication of property concepts in familiar cases with adjectives in languages such as English; the possessive morphosyntax provides the necessary semantic glue to relate a quality-type meaning to an ordinary individual. In the case of verbal possession, for example, the role of *tener*, *have*, etc. is to establish a possessive relation between a holder and state, which yields the same truth conditions as predication with an adjective having the same descriptive content. This idea is schematized in the derivation in (15), where *π* is the possessive relation (see Barker 1995:Ch. 2 for discussion). To have hunger is, on this view, to *possess some state of hunger*, or more technically as in (15c), for there to exist some state of hunger that an individual (in this case Kim) stands in the possessive relation to:^5^

(15)
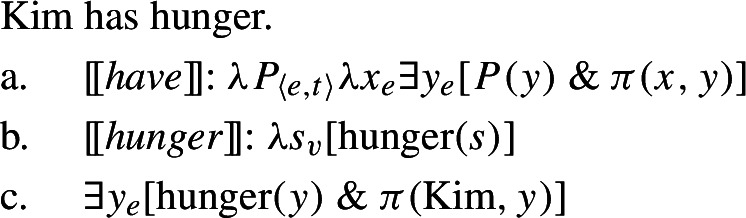

What such constructions all share, despite differences in the precise morphosyntactic manifestation of possession and the corresponding compositional details, is that they involve possession at the sentential level, relating some abstract property concept meaning to an individual via some possessive construction, whether with a verb, an adpositional construction, etc. As alluded to above however, Menon and Pancheva (2014) likewise implicate the use of possession in property concept *word* formation—as part of root categorization—to which we now turn.

### Possession at the word level

Expanding on Francez and Koontz-Garboden’s work, Menon and Pancheva (2014) argue for the strong claim that, universally, property concept words of all categories are built on acategorial, quality-denoting roots. A result of this claim is that, semantically, these roots must *always* be related to individuals by possession in predication. Notably, this possession is sometimes overt and sometimes *covert*, such that it is not always transparent on the surface.

Much of Menon and Pancheva’s (2014) analysis rests on constructions which (they claim to) involve both overt and covert possession in Malayalam (Dravidian). On the one hand, overt possession is observable at the sentential level with so-called ‘Class 2’ roots, which occur in the Malayalam existential possession construction in order to predicate. The examples in (16) demonstrate that property concept predication in this class is identical to ordinary possessive predication.

(16)
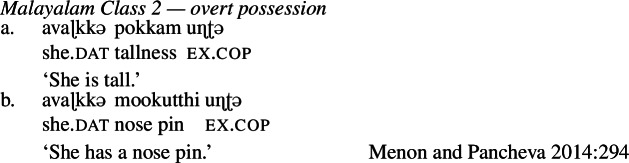

Examples of possession at the sentential level such as those in (16) are already familiar from above (cp. the Bisa in (14)). For details of the compositional analysis of these constructions, we refer the interested reader to Menon and Pancheva (2014), as exposition of the syntactic details, which are largely tangential to our concerns here, are intricate. The key points of their analysis are that (i) roots of this type are categorized by a nominalizer that does nothing to change the mass-type meaning of the root, and therefore (ii) the property concept nouns created by the categorization process require, reasonably in our view given the explicit morphosyntax of possession, possession in order to be turned into predicates of individuals. In this way, semantically speaking, constructions like (16) with Class 2 property concept words are treated in a manner parallel to the analysis of English possessed nominal property concept constructions articulated in (15).

While their analysis of Class 2 is fundamentally the same as the analysis of similar constructions analyzed by Francez and Koontz-Garboden, Menon and Pancheva argue that possession is also required at the *categorization* level in so-called ‘Class 1’ Malayalam roots, even though this is not reflected in the overt morphology of the language. In this case, their claim is that a covert verbalizer is required to turn a mass-denoting acategorial bound root into a predicate of individuals. Consider the examples in (17). By analogy with examples such as (17b), Menon and Pancheva offer an analysis of Class 1 property concepts as in (17a) as reduced relative structures (in which the suffix *-a* is a relativizer; see also Menon 2013):

(17)
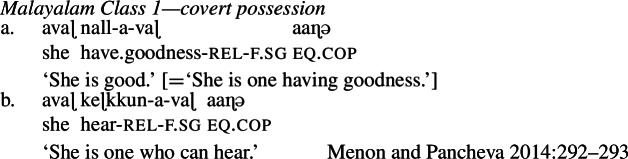

Their account of Class 1 property concepts (17) is that they are formed out of acategorial roots with Francez and Koontz-Garboden’s quality semantics along the lines of (18). (Class 2 property concepts have the same meaning, but are categorized as nouns.)

(18)
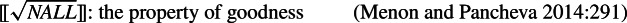

Class 1 roots must be verbalized before combining with the relativizing suffix, in view of the fact that the relativization structure is a predicate of ordinary individuals. Menon and Pancheva (2014) therefore argue that a null categorizer both verbalizes the quality-denoting root and introduces a possessive semantics:

(19)
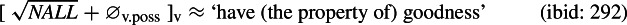

Class 1 and Class 2 roots have, then, the same kind of meaning—both have quality-type denotations. The difference between Class 1 and Class 2 *words*, is that while the former is categorized with possession, the latter is not. This being the case, words in the former class are predicates of individuals, while the latter have the kind of quality denotations that require possession to be introduced morphosyntactically in order to predicate of individuals.

This analysis is motivated by Menon and Pancheva’s a priori claim that all property concept words begin life as quality-denoting roots, and so require possession in predication to give rise to the truth conditions of adjectival predication in English. There is however variation in whether this possession is packaged with categorization (as with Class 1 roots) or not, in which case it must be introduced separately (as with Class 2 roots). Unlike for Francez and Koontz-Garboden, variation in the grammatical behavior of property concept words on this view has its source only in the functional domain, that is, whether overt possessive morphosyntax is available with a particular class, whether categorization and possession are packaged together, and in what functional heads are overtly realized; the quality-type meaning of property concept roots is, on Menon and Pancheva’s view, universal—and possession with them is too (with there being variation in exactly where the possession comes from). We now turn to property concept formation in Wá⋅šiw, which offers novel evidence against this view being generalized to all property concepts in all languages.

## Property concept verbs in Wá⋅šiw

We begin this section with further background on the Wá⋅šiw language and the sources of the data provided. We then lay out the empirical landscape of property concept verbs in the language before turning to the implications arising for possible variation in root meanings.

### Situating the language and the data

Wá⋅šiw (iso: was) is an Indigenous language spoken around Lake Tahoe (*dáʔaw*) on the California–Nevada border. Genetically, Wá⋅šiw is an isolate, though it is sometimes considered to be part of the proposed Hokan family (see Campbell (1997) and Mithun (1999) for discussion). It is a head-marking language with agglutinative morphology; much of the complexity in the language is found in the verb. The first (and only) grammar of this language comes from William Jacobsen’s (1964) UC Berkeley dissertation, which also lays out a pseudophonemic orthography. While revitalization efforts are active within the community, the language remains severely endangered with only a handful of native speaker elders alongside a growing population of L2 speakers. An overview of the history of linguistic work on the language along with a description of the current language situation can be found in Bochnak et al. 2023.

The data in this paper come from a variety of sources. These are: i) previously published work; ii) the Washo Archive, which is a corpus of examples currently housed at the University of Chicago;^6^ iii) tribal narratives, and iv) native speaker judgements from Hanink’s fieldwork — primarily with the late Adele James — which consisted of translation and truth-judgement tasks. Due to the language situation, the data available to the authors at present consists largely in these materials alone.

### Three morphological classes of property concept verbs in Wá⋅šiw

Property concepts in Wá⋅šiw take three different shapes. While all are verbal in category, they differ in morphological complexity.^7^ We henceforth refer to the property concept roots that appear in these different shapes in terms of classes: 1, 2, and 3.^8^

We begin with what we call Class 1 property concept roots, which behave identically to other intransitive verbs. These minimally require both prefixal subject agreement (Jacobsen 1964, 1977) and suffixal mood marking (Bochnak 2016; Bochnak and Hanink 2022), as exemplified in (20):

(20)
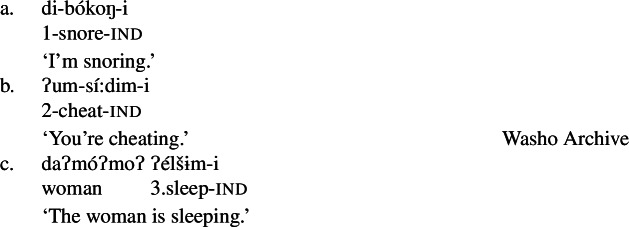

The examples in (21) demonstrate that Class 1 property concept verbs show the same morphological shape as the intransitive verbs in (20):

(21)

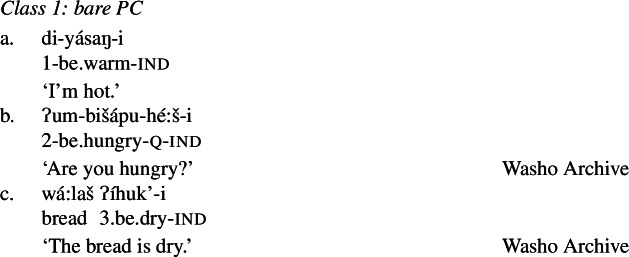

Class 2 property concepts require more complexity in their morphology. In this class, the suffix *-iʔ* is required in addition to the property concept root:

(22)
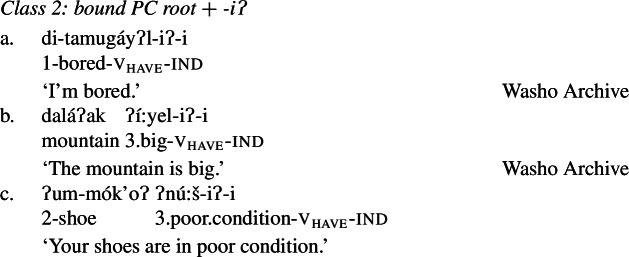

Foreshadowing our analysis somewhat, we gloss this morpheme as v_have_, though it is described as the ‘attributive-agentive’ suffix by Jacobsen (1964) (see Sect. 4.2).^9^ As stated, this suffix is obligatory for Class 2; property concept verbs in this class are ill-formed without it:

(23)

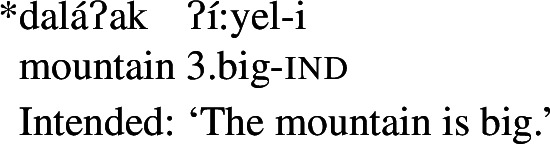

Finally, Class 3 appears to be a subclass of Class 2 insofar as property concept roots in this class likewise require the suffix *-iʔ*. However, Class 3 requires an additional morpheme: the prefix *ʔil-*. Roots in this class are also obligatorily reduplicated, a fact we return to in Sect. 5.2.^10^

(24)
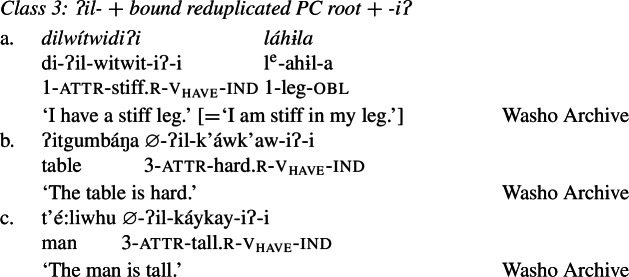

Roots in Class 3 are ill-formed as verbs if they are not flanked by both *ʔil-* and *-iʔ*:

(25)
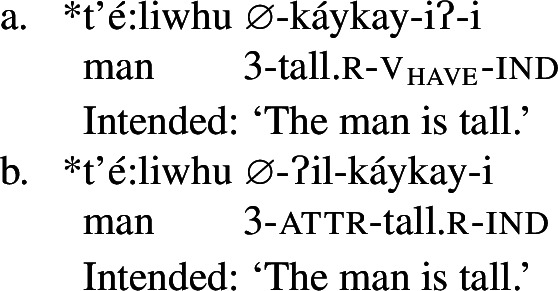

There is no flexibility about which class a particular property concept root belongs to, thus Class 2 roots are similarly ungrammatical alongside the prefix *ʔil-*, while Class 1 does not permit either affix. Table 1 offers a summary of morphological marking in verb formation by root class.

**Table 1:** Obligatory morphemes by property concept root class

	attr *ʔil-*	reduplication	v_have_ *-iʔ*	example	
Class 1	*	*	*	*yasaŋ*	‘hot’
Class 2	*	*	✓	*i:yel-iʔ*	‘big’
Class 3	✓	✓	✓	*ʔil-kaykay-iʔ*	‘tall’

We note that it is difficult to make semantic generalizations about which class a given property concept belongs to. For instance, these classes are not dictated by Dixon’s (1982) semantic classes. See the Appendix for a sample of property concepts in Wá⋅šiw organized by Dixonian category and morphological class, alongside some discussion of the difficulty in drawing generalizations emerging therefrom.

In the next section we lay out an account of variation in property concept root meanings that straightforwardly accounts for the morphological difference between Class 1 and 2 verb forms, before addressing the more complicated case of Class 3 in Sect. 5. The central claim of our analysis is that possession is only implicated in property concept formation when it is overtly realized—i.e., when *-iʔ* is present—necessitating variation in the meanings of Class 1 and Class 2 roots themselves.

## The basic contrast: Class 1 vs. Class 2

To implement our analysis, we follow Menon and Pancheva (2014) on the idea, couched in Distributed Morphology (Halle and Marantz 1993), that property concept words are derived from acategorial roots. The crux of the claim is that the *-iʔ* suffix in Wá⋅šiw verbalizes such a property concept root while simultaneously introducing a possessive relation. Crucially however, it does so only when the root is quality-denoting, in which case possession is required in order to create a predicate of ordinary individuals. In cases where *-iʔ* is absent, i.e., with Class 1 roots, this is because the root is already of the right type to predicate of ordinary individuals (i.e., a relation between sets of states and sets of individuals), rendering possession unnecessary and allowing for zero categorization just as with ordinary intransitive verbs in the language (cp. (20)).^11^

### Analysis of Class 1: Bare PC root

Recall that property concept roots in Class 1 as in (26) (repeated from (21)) form verbs that look identical to other intransitive verbs in Wá⋅šiw, i.e., they do not require any “extra” morphology.

(26)

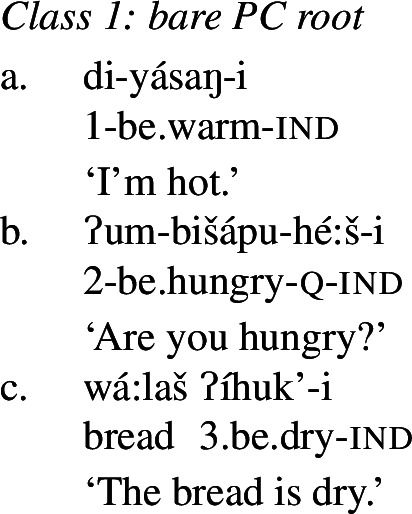

We adopt (the spirit of) Parsons (1990), Baglini (2015), Wellwood (2015, 2019), and others in assigning a Davidsonian analysis to property concepts. We treat Class 1 property concepts like other stative verbs under this approach: they denote relations between individuals and states. The root of the verb *ʔíhuk’* as in (26c) then denotes a relation between individuals *x* and dry states *s*:

(27)
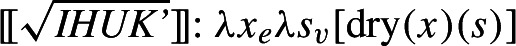

The proposition *wá:laš ʔíhuk’i* ‘the bread is dry’ as in (26c) will then be true if there exists some dryness state that stands in relation to some salient bread:^12^

(28)
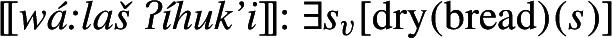

In summary, property concept roots in Class 1 denote relations between individuals and states, much like adjectives in English do (or can be assumed to do on a Davidsonian analysis). We take the (lack of) morphology of verbs formed from Class 1 roots at face value and submit that their roots are zero categorized in the same way as other intransitive verbs in the language; the covert verbalizer implicated therefore contributes no change in meaning. While this may be the obvious assumption to make given the shape of the morphology, this claim is further motivated by independent corroborating evidence laid out in Sect. 5.

### Analysis of Class 2: Bound PC root + *-i*ʔ

We now turn to Class 2 property concept roots such as those in (29) (repeated from (22)). This class can be differentiated from Class 1 in that the possessive suffix *-iʔ* obligatorily appears in addition to a bound property concept root.

(29)
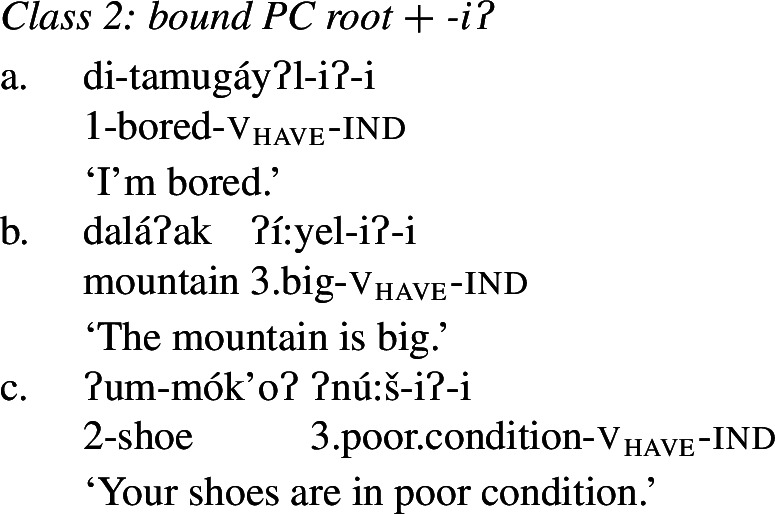

As stated above in the discussion surrounding (23), property concept roots in this class cannot appear without this suffix:

(30)
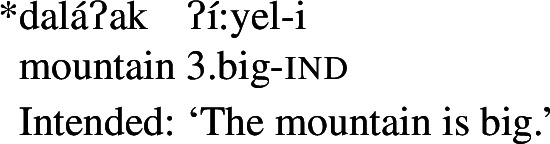

In what follows, we argue that we can understand the requirement for the *-iʔ* suffix straightforwardly if Class 2 roots do not denote relations between individuals and sets of states as is the case for Class 1 roots, but rather sets of states alone: roots in this class have the quality-type meaning that requires possession in order to form a predicate of ordinary individuals; a requirement fulfilled by possessive *-iʔ*.

As expected on such an analysis, and consistent with Francez and Koontz-Garboden’s findings, -*iʔ* is implicated in ordinary possession elsewhere in the language as what Jacobsen (1964:555) terms the ‘attributive-agentive’ suffix, which he describes as a denominal verbalizer that “derives verbs expressing the possessor of the underlying noun.” Jacobsen’s description arises from the fact that this suffix is used productively to express ordinary possession, as in the following:

(31)

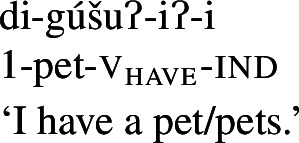

(32)
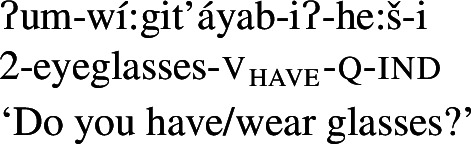

While we return to this construction in more detail in Sect. 6, one can see plainly from these examples that the *-iʔ* suffix contributes a possessive meaning such that a verb such as *gúšuʔiʔ* in (31) describes the set of individuals who stand in a possessive relation to one or more pets.^13^

Though Jacobsen (1964) does not relate the so-called attributive-agentive construction to the use of this suffix in property concept verb formation, we have seen above that, beyond cases of ordinary possession, it is also required in the formation of Class 2 property concept verbs.^14^ Drawing on Francez and Koontz-Garboden’s (2017) crosslinguistic observations, we can straightforwardly account for the parallel between the interpretation of this suffix in ordinary possession and its use in property concept formation. Similar to Francez and Koontz-Garboden’s (2017) treatment of Ulwa’s nominalizing -*ka*, we propose that *-iʔ* is a possessive verbalizer that introduces the possessive semantics necessary to turn mass-denoting roots into properties of individuals, at the same time that it turns an acategorial bound root into a verb.

From a semantic standpoint, we adopt from Francez and Koontz-Garboden (2017) the proposal that these roots have a quality meaning, as discussed above, articulating this in Davidsonian terms. On such a view, Class 2 roots in Wá⋅šiw then denote sets of states as in (33) (cp. the meaning of nouns such as *hunger* in (12)) for the property concept root *I:YEL* ‘big’ from (29b):

(33)
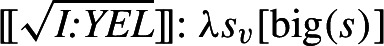

Because Class 2 roots do not denote sets of ordinary individuals, but sets of states, they must be possessed in order to be predicated of the resulting verb’s subject, as discussed above for the kinds of property concept nouns Francez and Koontz-Garboden examine. In the case of Wá⋅šiw, this is achieved by suffixing the verbalizing suffix *-iʔ*, which has the meaning in (34) (cp. (15)).

(34)
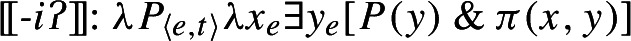

The result of composition is then as in (35), such that (35c) describes a set of individuals standing in a possessive relation to some state of bigness.

(35)

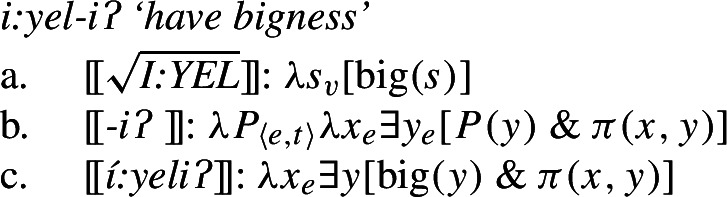

In a similar vein, we correctly predict that Class 1 roots will not permit categorization by the possessive suffix, as they are of the wrong type (〈*e*,〈*s*,*t*〉〉) to saturate the first argument of *-iʔ*, and more to the point already have the right kind of meaning to be directly predicated of ordinary individuals:^15^

(36)

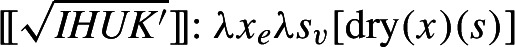

In summary, *-iʔ* is a verbalizer that is required to introduce a possessive semantics in cases where a property concept root denotes a set of states. It does not appear with Class 1, as roots in that class are already of the right type to act as predicates and therefore permit zero categorization as verbs. In Sect. 6, we return to this suffix and lay out a unified analysis of ordinary possession and property concept predication according to which *-iʔ* is a possessive light verb.

### Interim summary

In the preceding subsections, we have laid out a view in which Class 1 roots are directly categorized as verbs without any possessive semantics, while those in Class 2 are categorized by an overt possessive verbalizer, *-iʔ*. This state of affairs is a direct result of the difference in the lexical semantics of Class 1 and Class 2 roots—the former denote relations between individuals and states, while the latter denote only sets of states.^16^

The analysis thus far takes the morphology at face value in assigning different meanings to Class 1 and Class 2 roots. As a reviewer points out, another view of these data could simply be to list that certain roots are zero-categorized as verbs, while others require *-iʔ*. In our view, this misses a generalization: assuming that accidental homophony is to be avoided, and given that possessive morphosyntax is independently attested across a range of languages with property concept lexemes (Francez and Koontz-Garboden 2017; Hanink and Koontz-Garboden 2024) we believe it is undesirable to attribute the shape of class 1 and 2 property concepts verbs to morphophonological accident (see also related discussion in Sect. 5.3). However, while a WYSIWYG analysis has its conceptual advantages, it is clear that we have not—as yet—presented sufficient evidence to argue against the univeralist hypothesis for property concept roots put forward by Menon and Pancheva (2014), which would indeed avail itself of just such a morphophonological accident, with the lack of possessive morphology in Class 1 implicating a *covert* possessive categorizer for Class 1 roots (as in Malayalam Class 2), such that both Class 1 and Class 2 roots in Wá⋅šiw denote sets of states, with only Class 2 roots showing possession overtly. We argue in the next section that this cannot be the case, drawing on additional evidence that bears on this issue from Class 3 roots.

## Analysis of Class 3: *ʔil-* + reduplicated bound PC root + *-iʔ*

Recall from above that Class 3 roots are similar to Class 2 roots in that both require the suffix *-iʔ* to become verbs. Class 3 roots differ from those in Class 2 however in that they must also appear with the attributive prefix *ʔil-*, in addition to being reduplicated, as in (37) (repeated from (24)):

(37)
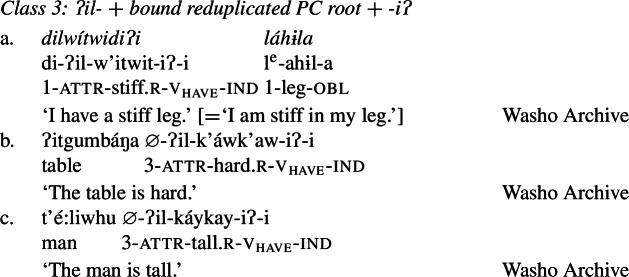

Despite this additional morphological complexity, we show that Class 3 roots in Wá⋅šiw have the same individual/state relation type of meaning as Class 1 roots, but require the prefix *ʔil-* for well-formedness reasons, which in turn leads to the necessity of *-iʔ*: due to a morphosyntactic quirk that is likely a historical artefact, these roots are not able to be zero categorized as verbs (cp. the English bound roots $\sqrt{\mathit{OPER}}$ of *operable*; $\sqrt{\mathit{STROY}}$ of *destroy*), nor are they of the right type to compose directly with the possessive suffix *-iʔ* in line with the Class 2 derivation. The consequence of this is that, despite their semantic equivalence to Class 1 roots, Class 3 roots must undergo two derivational steps in order to become verbal predicates.

More formally, we argue in what follows that the prefix *ʔil-* is semantically a type-shifter that takes a root with a Class 1 meaning and returns a set of states. For morphological reasons, *ʔil-* likewise triggers reduplication of the PC root. The crucial result is that a derived, but still bound form such as *ʔilkaykay* in (37) denotes a set of states, and thus requires the possessive semantics contributed by the *-iʔ* suffix in order to become a predicate of individuals. While this analysis may seem surprising at first glance, we provide in the next section independent evidence that Class 3 roots have the same meaning as Class 1 on the basis of their participation in resultative constructions. In Sect. 5.2 we return to the internal morphosemantic complexity of Class 3 items, having independently motivated the individual/state relation type meaning of roots in this class.

### Resultative bipartite verbs

Bipartite verbs (Jacobsen 1980), an areal feature of the northwestern United States (Sapir 1916; Jacobsen 1980; Mithun 2007; DeLancey 2009), are independent verbs formed from two otherwise bound morphemes (termed ‘initials’ and ‘finals’, respectively, according to the order they appear in). In Wá⋅šiw, the majority of verbs are bipartite in nature (Jacobsen 1980, Lemieux 2009; Bochnak and Rhomieux 2013). We discuss only resultative bipartite verbs here, of which property concepts are a necessary component (descriptions of a wider range of bipartite-verb types can be found in Jacobsen 1980 and Lemieux 2009; see also Bochnak and Rhomieux 2013).

Bipartite verbs figure into the analysis of property concept roots in two main ways. Firstly, property concept roots from Class 3 appear as finals in resultative bipartite verbs, to the exclusion of those from Class 2. Given that bipartite verb constructions distinguish between the classes of property concept roots in this way, if we have an otherwise motivated analysis of bipartite verbs generally, then they can offer a window into the analysis of property concept roots that do or do not appear in them. Secondly, and relatedly, bipartite verb contexts are the only environment in which Class 3 property concept roots appear on their own, without *ʔil-*, *-iʔ*, or reduplication. This being the case, they offer unique insight into the syntactic and semantic nature of the root in isolation. In what follows, we argue that this distribution lends support to an analysis of Class 3 property concepts in which they denote relations between individuals and states, just as Class 1 roots do. We also argue that it lends evidence counter to the universalist approach, as the fact that Class 2 roots are excluded from acting as finals in bipartite verbs can be captured if they have the kind of meaning we have proposed for them, which generates a type mismatch in bipartite verb contexts.

One of the core uses of bipartite verbs in Wá⋅šiw is in expression of change of state. Property concept finals figure prominently in these constructions, often with causative meaning. For instance, in the examples below, the final of the bipartite verb expresses a property concept state, while the initial expresses the agent’s manner of causing that state.

(38)
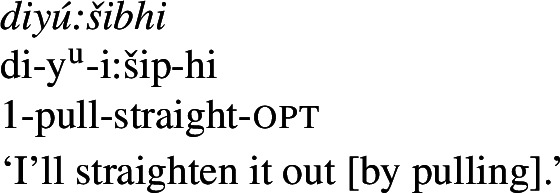

(39)
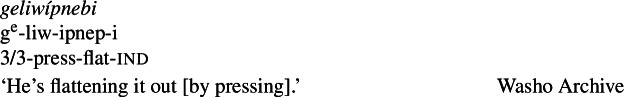

We believe that such constructions can be understood as a type of *resultative* construction (see Beavers 2012 for an overview) such as (40).^17^

(40) Kim hammered the metal flat.

There are many different kinds of resultative construction, and it is not our aim to give a survey here (see Beavers 2012:909–914). What matters for our purposes is that there is a variety in which the main verb describes a manner in which the state described by the result adjective (*flat*) comes about. Although there are many different analyses (see Beavers 2012), there is generally believed to be some compositional glue in constructions such as (40) in the form of causation, which does not seem to come from either the matrix verb or the result adjective on its own. How this meaning gets into the construction is a matter of dispute. Quite how this happens (if indeed it does) is tangential for our purposes, as we believe any analysis of resultative constructions applied to Wá⋅šiw bipartite verbs will have the same consequence we aim to highlight here, as our result is not tied to how causation is introduced: Class 3 property concept roots are acceptable in them, while Class 2 roots are not.

It is easiest to understand this in the context of some concrete analysis. So, although we do not believe that the details of any particular analysis impact on our claim (see fn. 20 for further discussion), we do believe it is important to choose one for expository purposes. We therefore lay out an analysis that is consistent with the facts of Wá⋅šiw bipartite verbs as we currently understand them (see Hanink and Koontz-Garboden (to appear)), which takes inspiration from the analysis of English resultatives to offer one possible analysis of the (for our purposes tangential) analytical question of how causation is introduced.

On this analysis of Wá⋅šiw bipartite verbs, we introduce causation constructionally, via a little v_cause_ head. We believe that this makes sense for bipartite verb constructions because, while they have a causative component, we do not believe there is evidence for that being introduced either by the initial or the final directly. In the case of the property concept finals under examination, it is uncontroversial that they are intransitive. Bipartite initials in general can participate in the formation of (causative) transitive verbs, as seen in the bipartite verb constructions under examination, but also intransitive ones, as illustrated for example by the change of state bipartite verb *dopoš* ‘burn gray’ in (41):

(41)
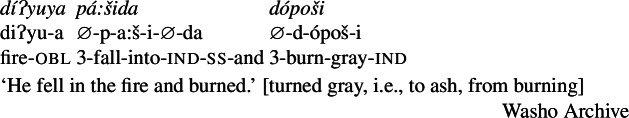

Given the absence of any additional morphological marking when a bipartite verb is used intransitively, as in (41), and transitively, as in (38) and (39), we assume that the transitivity, and indeed the causation, are coming from the bipartite verb construction itself. We therefore treat the manner component (e.g., *pulling, pressing*) characterized by the initial as the cause of the result state contributed by the property concept (e.g., *straight, flat*) final, with the causation introduced by v_cause_, which we take the initial to be a manner modifier of (see Lemieux 2009 for a related templatic proposal). Taking the bipartite verb *liwipnep* ‘flatten out by pressing’ as an example, we therefore propose (42a) and (42b) for the meanings of the initial and final, respectively:

(42)
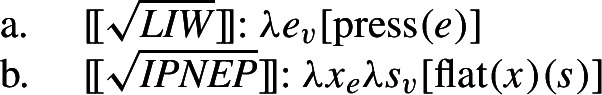

We assume, in a manner parallel to what we assume for the causative entailments, that change of state entailments are also introduced by a functional verbal head, following work by Embick (2004), Ramchand (2008), Alexiadou et al. (2015), Folli and Harley (2020), among others.

(43)
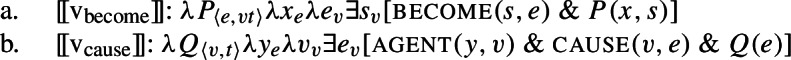

Putting these pieces together in the kind of syntactic approach they presume, we arrive at the following configuration and interpretation for a bipartite resultative such as (39), repeated in (44a) (prior to word formation; cp. Liu’s (2021) analysis of Mandarin V-V resultatives):^18^

(44)

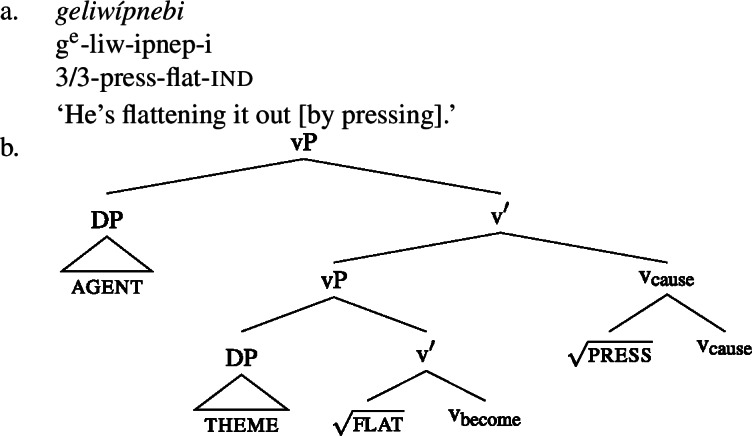

The property concept root in (42b) and the v_become_ in (43a) compose via function application to yield a relation between individuals and straightening events. After composition with the theme argument by function application, we generate the denotation for the vP in (45). The denotation for the v_cause_, for its part, is also generated through function application, taking the root meaning in (42a) and the v_cause_ meaning in (43b) and composing them to yield the denotation for v_cause_ in (45b). The vP and the v_cause_ compose with one another through event identification to yield the denotation for the v′ in (45c).

(45)
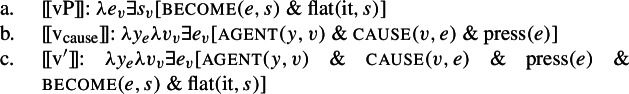

After the external argument is added and entered into the composition, and the Davidsonian argument is existentially closed, we derive the logical form in (46), which yields the right truth conditions for (39).

(46)

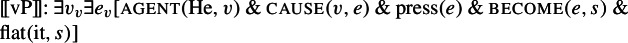

This analysis derives the attested truth conditions for the kinds of resultative bipartite verb constructions we find in the language. And as stated above, these are crucially *Class 3 roots*. This makes sense on the analysis of bipartite verb constructions that we have developed here on the assumption that these roots denote, as we have argued above, relations between individuals and states. This is so partly, and crucially, because v_become_ relates the event of change to a relation between an ordinary individual and a state which that individual is a holder of. Class 2 roots, however, do not have this kind of denotation. While Class 3 roots relate an individual to some state, Class 2 roots denote predicates of states themselves, and are therefore of the wrong type to compose with v_become_ in the first place. Instead, in order to be turned into relations between individuals and states, they must appear with the possessive suffix *-iʔ*. But the bipartite verb syntax does not accommodate this morphology, and because of this, and because they have the wrong meaning in the first place, they cannot appear in this construction.

We exemplify this claim with the hypothetical, but importantly *unattested* (which we mark with ‘˜’), bipartite verb in (47), composed of the class 2 final ‘big’ and the initial ‘pull’:^19^

(47)
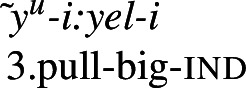

If the Class 2 root *i:yel* ‘big’ were to appear in a bipartite verb construction like (47), it would have the structure in (48), parallel to what we have seen above for the bipartite verb constructions which are indeed attested on the basis of Class 3 finals.

(48)

The crucial part of the structure in (48) is in the lower v′, where *i:yel* and v_become_ would have to compose. As argued above, the semantics of the possessive suffix *-iʔ* and the meaning that Class 2 roots get when they appear with *-iʔ* motivate a meaning for Class 2 roots as predicates of states, giving *i:yel* the denotation in (49a). Further, we have assumed a denotation for v_become_ as in (49b), on which it takes an individual/state relation as an argument, returning a relation between individuals and events of change into the state described by that predicate undergone by the individuals in question. This relation is of type 〈*e*,〈*v*,*t*〉〉; but Class 2 roots are of type 〈*v*,*t*〉 leading to a straightforward type-mismatch, from which it follows that Class 2 roots cannot appear in this construction.

(49)
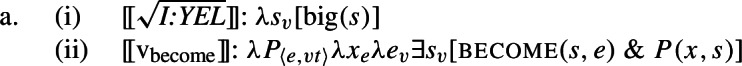

The key observation is that the meaning we have independently motivated via the possessive suffix for Class 2 roots in Sect. 4.2 makes it so that these roots are predicted not to occur in the bipartite verb construction. And indeed, these roots are not found in this construction.^20^

As a final supporting observation, we note that there is related evidence from this construction for our claim that Class 3 roots have the same kind of meaning as Class 1 roots, leaving Class 2 roots the odd ones out. In limited cases, Class 1 roots may also appear with initials in bipartite constructions. In the resultative type, for example, the Class 1 property concept root *ihuk’* ‘dry’ may also appear in this position.

(50)
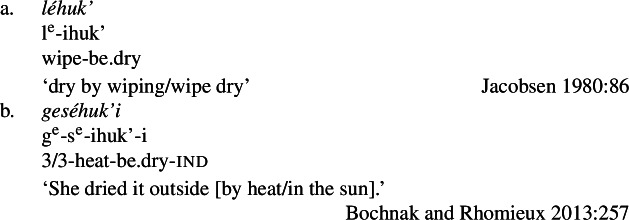

This shows that, in addition to Class 3 roots, Class 1 roots are felicitous as the result component of resultative bipartite verbs, to the exclusion of Class 2. We can understand this if only the latter are mass-denoting, unlike roots belonging to Classes 1 and 3, but not if we treat the semantics of property concept roots in all three classes homogenously, as Menon and Pancheva’s hypothesis would dictate.

### Back to *ʔil-*

We are now in a position to more explictly evaluate the role of the attributive prefix *ʔil-*. As background, there are three aspects of Jacobsen’s (1980:86) discussion of *ʔil-* that must be borne in mind. Jacobsen claims: (i) that this prefix is actually part of a circumfix alongside the attributive suffix, the circumfix being an allomorph of the attributive suffix on its own; (ii) at the same time as (i), that *ʔil-* is a bipartite initial comprising its own class ‘descriptive of appearance’ (see Bochnak 2013:Ch. 5 for further discussion); and (iii) that this prefix selects for a reduplicated root.^21^

There are several additional facts that we add to Jacobsen’s which must be borne in mind when considering how to analyze this morphology. Firstly, while Class 3 roots are obligatorily reduplicated in the context of the prefix *ʔil-*, this is *not* the case when they appear as finals in bipartite verb constructions. The examples below offer minimal pairs contrasting Class 3 roots prefixed by *ʔil-* ((51a), (52)[a]) with those prefixed by a true bipartite initial ((51b), (52)[b]) ((51b) repeated from (38)).

(51)

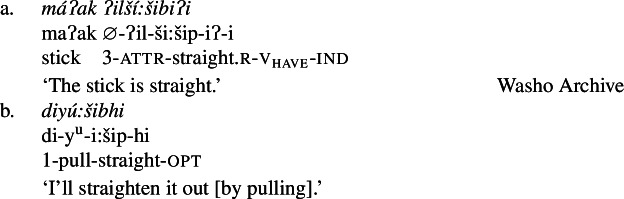

(52)
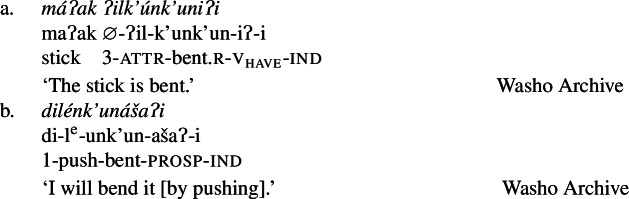

It is also worth highlighting the differences, particularly in the context of Jacobsen’s claims, between *ʔil-* and ordinary bipartite initials, particularly in that the former has no identifiable meaning on its own (cp. *y*^*u*^*-* ‘pull’; *liw-* ‘press’). Instead, it appears simply to be used in contexts in which the property concept predicate is used to describe the subject of the verb (as observed by Lemieux 2009), rather than being used as a dependent form (as in bipartite verbs).

Relevant also is the status of reduplication with Class 3 roots in ordinary predication. Traditional analyses, beginning with Jacobsen 1964, treat the type of reduplication with vowel-initial roots as in (51a) and (52a) as a partial one (Jacobsen 1964; Werner 1970; Broselow and McCarthy 1983; Urbanczyk 1993; Yu 2005), in which the onset of the final syllable is copied (53). In what follows, we frame the discussion in terms of this traditional view.^22^

(53)
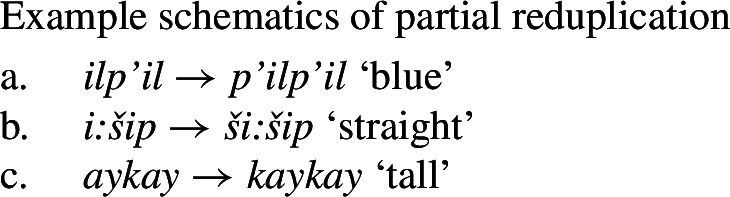

Taking the requirement both for the prefix *ʔil-* and for reduplication into consideration, there are several questions that an analysis of this construction would ideally answer. Firstly, why is the root reduplicated? Secondly, what is the role of *ʔil-*?

As mentioned above, our proposal is that *ʔil-* is a type-shifter. This analysis adopts different aspects of Jacobsen’s description of this construction, departing from him in other ways. Specifically, we depart from Jacobsen in assuming that that the morphology is *not* a circumfix, but rather that *ʔil-* is a prefix in its own right. We follow the spirit of Jacobsen’s discussion on reduplication, however, in assuming that the reduplication in the *ʔil-* construction is semantically meaningless and pure morphology required in this construction. Crucially, the treatment of the verbalizing suffix *-iʔ* that we have motivated above remains the same, namely that it is possessive and creates a predicate of ordinary individuals out of a quality-denoting linguistic object.

Given the claim that Class 3 roots denote state/individual relations, the question that any analysis of these forms must answer moreover is ultimately how possession (implicated by the requirement for *-iʔ*) is involved in order to get the right kind of meaning to create a predicate of individuals. This is a particularly urgent question in the context of the *ʔil-*prefixed forms, given that these are found only with Class 3 roots such as (54) (repeated from (51a)), which we have argued denote relations between individuals and states (55) on the basis of their appearance in resultative bipartite verb constructions discussed in Sect. 5.1.

(54)

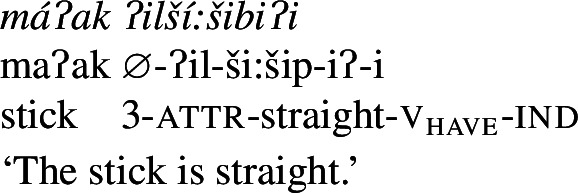

(55)
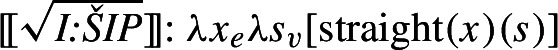

Consistent with the analysis of the verbalizer *-iʔ* we have motivated above, this is the *wrong* kind of meaning for it to take as an argument, as seen in (34) and repeated in (56).

(56)

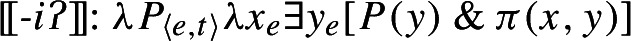

We believe there is a ready-made solution to this conundrum which lies in answering the question of what the role of *ʔil-* in this construction is. Our analysis is that it is precisely the prefix *ʔil-* that is giving the root the kind of meaning required in order to be an argument of the possessive suffix: It type-shifts an individual/state relation type meaning into a quality-type meaning, which can be possessed. Such a denotation is articulated for *ʔil-* in (57), where ^▽^ is a function (inspired by Chierchia’s (1984) ^∩^ operator, but different in many ways), which takes a relation P of type 〈*e*,〈*v*,*t*〉〉 and returns P’s range.^23^ In other words, it takes an individual/state relation and returns the set of states that underly the relation.

(57)
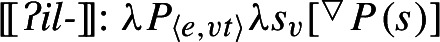

It seems hardly accidental that Class 3 roots in typical bipartite verb constructions, as in (38) above, appear in their bare, nonprefixed root form, a use which, as discussed above, provides independent evidence for our claim that these roots have the type of meaning in their bare form articulated in (55) (by contrast with the quality-type Class 2 roots, for example). It is also only, as Jacobsen observed, when they are found in the *ʔil-*prefixed construction with a stative meaning that they must be reduplicated. Following Jacobsen, reduplication on this analysis is then purely a morphological requirement of *ʔil-*. This has the consequence that the reduplicated root, taking *iši:p* ‘straight’ as an example, has the same meaning as the bare root, i.e., it denotes a relation between individuals and states, as in (58):

(58)
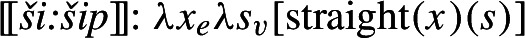

The prefix then composes with the reduplicated root to create a predicate of states, as in (59). This operation thus yields a quality type meaning—a predicate of straightness states in the case of (58)—with the same type of meaning as Class 2 roots, but in this case derived, by contrast with the Class 2 ones which have this kind of meaning inherently.

(59)
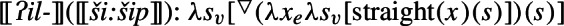

The result of this type shift is a meaning that can be possessed by the suffix *-iʔ*, which on this analysis remains an independent suffix consistently across classes.^24^ This suffixation turns the predicate of states into a relation between ordinary individuals and those states, in the case of (60), between ordinary individuals and straightness states, i.e., those individuals that possess some straightness state, which is what (60) says, albeit in cumbersome notation which is an artifact of the quality-type meaning being derived via ^▽^ as the semantic reflex of prefixation by *ʔil-*:

(60)
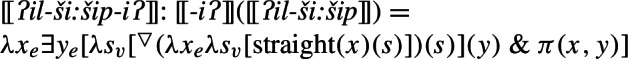

That a predicate like (60) can be predicated of an ordinary individual to yield the right truth conditions for a sentence like (54) should be obvious, and we assume it has a syntax on a par with predication with Class 2 roots, the only difference between them being that it is not a simple root that appears as the argument of the possessive verbalizer *-iʔ*, but rather the *ʔil-* prefixed reduplicated root, for the semantic reasons discussed, thereby adding the extra layer of complexity.

Crucial to this analysis is that *ʔil-* composes (structurally speaking) with the reduplicated root prior to the possessive suffix *-iʔ*, which takes the entire *ʔil-* prefixed root as an argument. The alternative syntax whereby the possessive *-iʔ* suffix takes the reduplicated root as an argument directly (or indeed the bare root) would fail to compose semantically, as neither form has the predicate of states type meaning that the suffix is looking for. On this analysis it is only after prefixation by *ʔil-* that such a meaning is generated. This analysis correctly predicts that only Class 3, and not Class 2, appears with the prefix (and hence reduplicated), on the grounds that Class 2 roots can compose directly with the possessive suffix *-iʔ*, while Class 3 roots cannot.

What remains an open question is what exactly motivates the Duke-of-York type derivation (Pullum 1976) we see, where the root meaning is type-shifted from an individual/state relation only to be shifted back again. We speculate that Class 3 roots are prisoners of morphological well-formedness conditions. The roots themselves have the right kind of meaning to be predicated of individuals directly, without the possessive suffix. In this way, they are the same as Class 1 roots. They crucially differ however in that, while Class 1 roots can be zero categorized as verbs, Class 3 roots—which only ever appear as dependent forms—cannot, and therefore require the prefix *ʔil-* to ultimately become well-formed.^25^

This is because, as far as we can tell, the language has no other morphology that can verbalize such a root without altering its meaning. While in the context of bipartite verbs this can be done with an initial, these do not allow for the creation of a stative word with only the meaning of the root. The only option that Wá⋅šiw grammar seems to have for creating a stative predicate out of these roots is via the possessive suffix, which itself requires a predicate of states, rather than a state/individual relation, as an argument. On this analysis, that is precisely the semantic role of *ʔil-*, which from an individual/state relation returns a set of states that can then be semantically possessed.

In sum, this analysis correctly captures the fact that Class 3 roots, by contrast with Class 2 roots, appear with the prefix *ʔil-*, which is treated on this analysis as a type-shifter. On the other hand, Class 1 roots avoid the Duke-of-York derivation altogether because they are permitted to be zero categorized as verbs directly, and so do not need to be made into one.

### Interim summary

The aim of the preceding section has been to show that Class 3 roots, like those in Class 1 and to the exclusion of those in Class 2, denote relations between individuals and states. While our analysis of Class 1 and Class 2 roots, which takes the presence or absence of possessive morphology at face value, does not on its own counterexemplify a universalist view of property concept roots, the concomitant behavior of all three classes in bipartite verb contexts shows that they cannot all be quality-denoting. If they were, we would predict all three class types to be possible in bipartite resultative verbs, contrary to fact. Instead, the exclusion of Class 2 from precisely this context, along with the appearance of Class 1 and 3 roots in their purest form, supports a view in which only the Class 2 are quality-denoting, entailing a state of affairs in which property concept roots show variation in their lexical semantics. This view is moreover supported by the superficial morphological differences between the classes in their ordinary predicative uses, with Class 1 lacking the possessive verbalizer *-iʔ*, Class 2 taking it directly (consistent with a quality-type meaning), and Class 3 taking it only after undergoing additional morphological alteration. To put it differently, we have started with a morphological contrast: Class 2 take the possessive verbalizer *-iʔ*, while Class 1 do not, and Class 3 take it only in combination with additional morphology. This morphological contrast correlates with the bipartite verb gap: that Class 2 do not appear in such constructions, while Class 1 and 3 do, a gap which makes sense in the context of a compositional analysis of this construction if the contrast in root meanings independently motivated by the possessive verbalizer morphology holds.

Before moving on, we address the high-level question raised by an anonymous reviewer, which our observations invite: why do some roots denote relations between individuals and sets of states, and others sets of states alone? On our analysis as it stands, this difference is not predictable. We do not make any predictions about which kind of meaning which root will have; we simply make this determination for Wá⋅šiw on the basis of what the grammar tells us in the form of the grammar of possession and resultative bipartite verbs in the language. It could well turn out that roots with particular kinds of Dixonian meanings are more predisposed than others towards having a certain type (type-theoretically speaking) of meaning. This strikes us as an intriguing question for future crosslinguistic research, but not one that we are in a position to offer further reflections on here.

Another question raised by a reviewer in relation to our claimed explanation merits discussion here as well, namely whether there aren’t alternative possible analyses that the data might be consistent with. We sympathize with this concern: we believe that in general it is the case that available linguistic data underdetermines analyses, and that this is especially the case when studying languages like Wá⋅šiw, where there is a paucity of data available by comparison with most languages, not least very well-studied ones. The reviewer questions whether, for example, it might not be the case that Menon and Pancheva’s conjecture holds for Wá⋅šiw, and that the patterns of use we observe with *-iʔ* are simply a morphophonological accident. While we have tied Class 2’s appearance with *-iʔ* in predication with its absence in the bipartite resultative construction, on such an analysis, the reviewer suggests, Class 2’s absence would also be accidental. We cannot, of course, rule out such an analysis. At the same time, we also believe that the general modus operandi of linguistic analysis is that explanation is to be preferred over accident when an explanation is available. When that is coupled with the fact that the behavior of *-iʔ* qua possessive fits in with a known crosslinguistic generalization regarding the nature of the lexical semantics of property concept lexemes, as discussed at length by Francez and Koontz-Garboden (2017), we believe our explanation of the behavior is preferable.

## *-iʔ* as a possessive light verb

Given that the majority of property concept roots in Wá⋅šiw require possessive morphology in predicative contexts, we now turn to evaluate the suffix *-iʔ* in more detail. We argue that the characteristics of this suffix support a view in which it is the realization of a functional light verb (Johns 2007, 2009; Yuan 2018), into which either a nominal (in the case of ordinary possession) or a bare root (in the case of property concept formation) must incorporate (Johns 2007, 2009).

### *-iʔ* in ordinary possession

In light of the proposed analysis of property concept word formation above, the remaining piece of the puzzle is now to establish precisely what the syntactic status of *-iʔ* is. We have argued that what Jacobsen (1964) termed the ‘attributive agentive’ suffix *-iʔ* is a verbalizer that introduces a possessive semantics.^26^ Evidence for a treatment of the *-iʔ* suffix as a possessive verbalizer in particular comes from its appearance in ordinary possessive predication, as described in Sect. 4.2: Outside of property concept verb formation, this possessive function can be clearly observed in examples such as those in (61).

(61)
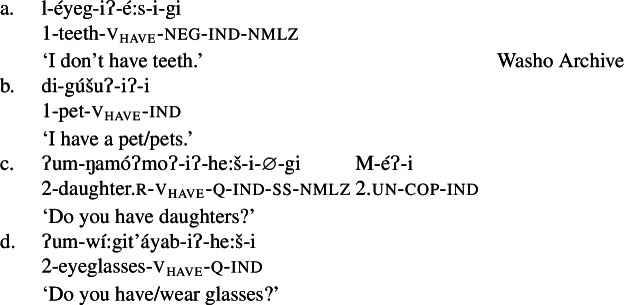

Indeed, this construction is the primary means of possessive predication in the language, used to express both notionally alienable (61a) and inalienable (61b)–(61d) possession.^27^ While ordinary possessive predication is not the primary focus of this paper, understanding how *-iʔ* works in this construction supports our claims regarding its function in property concept word formation.

Toward an analysis, we observe that a similar possessive construction is found in Hiaki (Uto-Aztecan) (Jelinek 1998; Harley 2002), albeit differing from Wá⋅šiw in that there is no overt possessive morphology despite the clearly possessive interpretation.

(62)
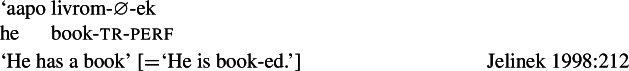

Jelinek (1998) analyzes these constructions syntactically, proposing that an NP moves into an empty verbal position, which she terms a transitivizer. In a similar vein, though addressing a more diverse set of facts, Johns (2007) argues that noun incorporation in Inuktitut is found (only) with a range of light verbs (see also Yuan 2018), one of which is *-qaq*, shown in (63), which expresses possession:^28^

(63)
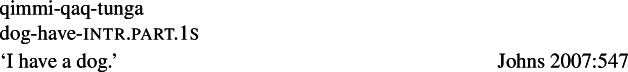

Evidence that these incorporating elements are light verbs rather than lexical verbs comes from the fact that incorporation is obligatory; such elements cannot stand on their own as verbal predicates. The same is of course true for Hiaki (as the morpheme is null), and holds for Wá⋅šiw as well: the suffix *-iʔ* cannot stand on its own as a verb (cp. (61b)):

(64)
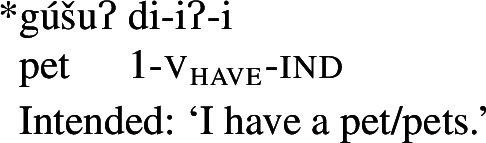

In the case of Inuktitut, Johns (2007, 2009) argues that what “incorporates” into v_have_ is a root rather than a lexical NP, such that these constructions are not an instance of incorporation per se, but rather *categorization* (see also Massam 2009 for discussion). She notes that this proposal is in line with Sapir’s (1911:254) observation that these “verbal elements are not verb stems but verb-forming affixes.” While subsequent work by Compton (2013) shows that this root incorporation is untenable for Inuktitut (based for instance on the fact that full DPs may incorporate), Yuan (2018) maintains that the trigger for incorporation of lexical NPs in the language remains its inventory of functional light verbs along the clausal spine.

We build on this idea in our analysis and propose that *-iʔ* is a light verb, v_have_, that triggers obligatory incorporation of a nominal.^29^ That ordinary possession is the result of true NP incorporation is supported by the fact that this process may leave stranded modifiers behind (65c).^30^ That these modifiers modify the noun directly prior to movement moreover is seen through the fact that they exhibit the suffix *-w* where appropriate, a plural concord marker that only appears with animate nouns (Hanink 2021) (as in (65b and c), cp. inanimate *gumʔé:be* ‘day’ in (65a)).

(65)
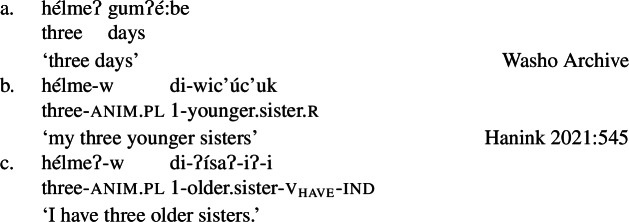

Adopting the idea that concord is the result of agreement with functional heads within the extended nominal projection (e.g., n, Num; Ritter 1991, 1993; Kramer 2009), the noun must be categorized prior to movement in order for this concord to be possible. We therefore arrive at the following schematic for an example such as (65c):^31^

(66)

Assuming that movement of the head is not relevant to interpretation, v_have_ selects for the entire nominal as its complement prior to movement. Adopting a standard semantics for cardinalities according to which they are restrictive modifiers (i.a. Bartsch 1973; Hoeksema 1983; Chierchia 1985), semantic composition is as follows, assuming the now familiar interpretation for *-iʔ*.^32^

(67)
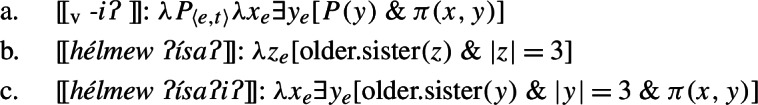

To summarize, we analyze the suffix *-iʔ* in Wáːsiw as a light verb v_have_. In the case of ordinary possession, an already categorized noun undergoes incorporation into this head, forming a verb that expresses possession of that noun. In the next subsection, we revisit Johns’s (2007) root incorporation analysis in our analysis of *-iʔ* in property concept word formation.

### *-iʔ* in property concept word formation

We argued above in Sect. 4.2 that *-iʔ* performs a categorizing function in the case of Class 2 roots. The primary evidence for this claim is that roots in Class 2 are bound as in (68) (repeated from (22b) and (23)), and therefore do not form standalone verbs (or indeed any other category):

(68)
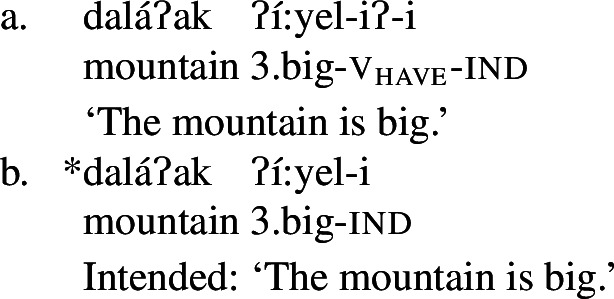

A similar state of affairs was shown to be the case for Class 3 in Sect. 5, whose roots are however required to combine with *ʔil-* prior to categorization by *-iʔ*; the only class for which v_have_ is not required is Class 1, whose members are able to be categorized as verbs without an overt verbalizer. We therefore propose the following structure for *-iʔ* in its function as a root categorizer:^33^

(69)

The purpose of *-iʔ* is two-fold: 1) to categorize the root and 2) to introduce a possessive semantics. Beyond the bound nature of the roots it combines with, evidence that *-iʔ* is a categorizer comes also from the fact that it is always the closest affix to the property concept root. For example, it appears inside other verbal morphology, such as the aspectual suffix *-ašaʔ* and the inchoative suffix *-etiʔ* (70):^34^

(70)

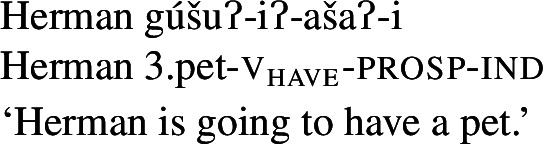

(71)

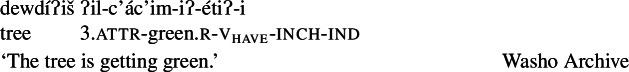

In this sense, Wá⋅šiw exhibits stacking of light verbs, exemplified even more acutely in (72) which demonstrates the sequence v_have_-v_become_-v_cause_:

(72)
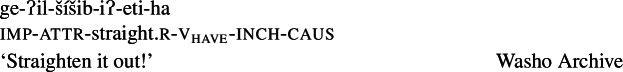

Before concluding, we note that this state of affairs is not necessarily limited to Wá⋅šiw: preliminary evidence for a similar verbalizer with a possessive semantics is found in Huitoto (Huitotan), as discussed by (Francez and Koontz-Garboden 2017:28) (see also Hanink and Koontz-Garboden 2024). In the following examples (apud Minor et al. 1982) the suffix -*re* is found in both ordinary possession and in property concept predication.^35^ While Minor et al. (1982:49) intimate that *-re* is a verbal categorizer, there is limited data supporting this conclusion; we include these examples here to highlight a potential avenue for crosslinguistic comparison.

(73)

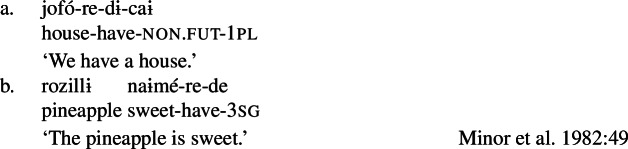

In summary, the suffix -*iʔ* in Wá⋅šiw is a light verb v_have_ into which either a nominal, as in the case of ordinary possession, or a root, as in the case of property concept formation, must incorporate. These restrictions are derived semantically, determined by the type of the root: this light verb may only compose with meanings of type 〈*e*,*t*〉, ruling out direct composition with Class 1 roots, and Class 3 roots on their own without *ʔil-*.

## Conclusion and outlook

In the preceding sections we have articulated a three-way contrast in the morphosyntactic behavior of property concept verbs in Wá⋅šiw, summarized in Table 1 and repeated in Table 2.

**Table 2:** Obligatory morphemes by property concept root class

	attr *ʔil-*	reduplication	v_have_ *-iʔ*	example	
Class 1	*	*	*	*yasaŋ*	‘hot’
Class 2	*	*	✓	*i:yel-iʔ*	‘big’
Class 3	✓	✓	✓	*ʔil-kaykay-iʔ*	‘tall’

We have shown that the contrasting behavior embodied in Table 2 motivates contrasting meanings for the roots belonging to the different classes. Class 2 property concepts require the possessive verbalizer *-iʔ* because they denote predicates of states, which cannot themselves be predicated of ordinary individuals without possessive morphology, consistent with Francez and Koontz-Garboden’s crosslinguistic observations regarding the use of possessive morphology in property concept predication. Class 1, by contrast, predicate directly, as other verbs do, because roots in this class are of a type for which this is possible, denoting individual/state relations. Class 3 are victims of morphology, having the right kind of denotation to predicate directly, but are unable to do so in virtue of their root type, which cannot be directly verbalized. Because of this, they appear with prefixes and suffixes that can turn them into individual/state relations. These claims about root meaning, moreover, are independently supported by behavior in the bipartite verb resultative construction, which requires bare roots, and therefore serves as a window onto their meanings. Our meaning claims are therefore supported by the fact that a reasonable compositional semantics for this construction requires of the roots that they can directly predicate of ordinary individuals, something possible for Class 1 and Class 3, but not Class 2. It is crucial, therefore, that we observe just Class 1 and Class 3 in bipartite resultative verb constructions, but not Class 2, precisely as predicted independently by the semantics that the morphosyntactic behavior in property concept predication predicts.

While the contrasting property concept root meanings we have argued for are straightforwardly motivated by the facts of Wá⋅šiw grammar, their theoretical consequences are significant. Much recent work at the syntax–semantics interface derives words in the syntax. It is therefore tempting to assume that words with the same meanings across languages are derived from the same underlying semantic core. Menon and Pancheva (2014) articulate this idea explicitly in the domain of words with Dixon’s (1982) property concept meanings, developing an analysis in the context of Distributed Morphology on which all such words, in all languages, are derived from a quality-denoting acategorial root. This root must then compose, via either categorization or other functional material, with possession in order to predicate of ordinary individuals, consistent with Francez and Koontz-Garboden’s analysis that only then can such meanings be predicated of ordinary individuals. On this view, crosslinguistic variation in the grammatical behavior of property concept words arises not through differences in the meanings of property concept roots, but rather through differences in the semantics of categorizers (whether they have a possessive semantics or not) and the inventory of functional heads (determining the syntax of possessive predication). By contrast with Menon and Pancheva’s universalist theory of root meaning, Francez and Koontz-Garboden develop a theory instead where property concepts vary in meaning at their core, with only those that trigger overt possession having a quality-type meaning, and those that do not, having the kind of meaning standardly assumed in the formal semantics literature on adjectives (whether degree-based, as in Cresswell 1977 and those following him, or supervaluation-based, as in Kamp 1975; Klein 1980 and those following them). In this way, variation is tied directly to the lexical semantics of the property concept roots themselves.

In this paper, we have considered these competing hypotheses of variation in this domain by offering an existence proof of sorts which shows that Menon and Pancheva’s strong theory cannot be correct. We have shown that there are language-internal arguments from Wá⋅šiw demonstrating that the language has property concept roots with different meanings, whose grammatical behavior can be understood with reference to that difference. A reviewer wonders how a learner would settle on the contrast in meaning that we have argued for, and we believe it is precisely this grammatical behavior that would point a learner to this contrast. Once they learn that that *–iʔ* is possessive, following the arguments in Francez and Koontz-Garboden (2017), the learner will be forced to the conclusion that roots appearing with it have a kind of meaning consistent with it, while those not appearing with it do not. This conclusion will be reinforced by behavior in the bipartite resultative construction. It is true that the learner could instead posit some null possessive morphology and assume, consistent with Menon and Pancheva, that Class 1, 2, and 3 all have an abstract mass type meaning. But the learner would then run into trouble with the bipartite resultative verb construction, and would therefore be forced to reevaluate that (erroneous) meaning assignment.

Whilst we do not believe, therefore, that this part of Menon and Pancheva’s theory is correct, we do believe other aspects of it are indeed supported (contrary to claims in Francez and Koontz-Garboden 2017). Indeed Hanink and Koontz-Garboden (2024) show that there is unequivocal evidence that possessive categorizers exist across the major categories, as they claim. What we have shown here is simply that the strong, universalist aspect of their root meaning claim is falsified by data from Wá⋅šiw. While it is clear that there are languages with property concept words that are derived from bound quality-denoting roots categorized with a categorizer with possessive semantics—indeed we have argued for precisely this in the case of Wá⋅šiw Class 2—it is not the case for all property concept words in all languages, as the strongest version of their hypothesis would have it.

While we agree with Menon and Pancheva that inventories of functional vocabulary are no doubt a source of crosslinguistic variation, our results also point to roots themselves as a source of variation. In this way, our results push back somewhat against recent work that argues wholly against variation in the semantics of open-class items as a source of crosslinguistic variation. While Talmy (1985), for example, argued that languages vary in whether motion verbs have manner or path conflated with motion, more recent work by Beavers et al. (2010) argues that much of the variation observed in the literature on motion predicates can be pinned on variation in functional inventory. Similarly, many have questioned Chierchia’s (1998) central claim that languages vary in whether their nouns have basic mass or count denotations (see Dayal 2022 for an extensive discussion and references). And the situation is similar for recent evaluations of Beck et al’s (2010) Degree Semantics Parameter, which has it that open-class property concept words vary crosslinguistically in whether they have a semantics of degree or not, with Bochnak et al. (2020) arguing, again, that the observed variation can be better explained with reference to functional vocabulary. Nevertheless, our results here suggest a nuanced conclusion—that variation in the lexical semantics of open-class items, while certainly not the sole source of variation in morphology and syntax, does retain a role in explaining variable behavior both within and across languages.

## Appendix: Sample of property concepts in Wá·šiw

Table A1 offers a sample of 70 property concept stems in Wá·šiw.^36^ These stems are organized both by Dixonian category, as well as by the class number schematic we have followed in this paper (1, 2, and 3). None of the categories are meant to be exhaustively represented. Note that class 3 property concept stems are listed in their reduplicated form.

**Table A1:** Sample of 70 property concepts in Wá·šiw

Category	Class	Stem	PCL	Source
Age	1	MiLe	old	Washo Archive
	2	ešlut’	young	Washo Archive
Color	3	leleg	red	Washo Archive
		p’ilp’il	blue	Washo Archive
		popo	white	Washo Archive
		šošoŋ	brown	Washo Archive
		ʔyɨŋʔyɨŋ	varicolored	Washo Archive
Dimension	1	beheziŋ	small	Washo Archive
		wgohat	wide	Bochnak 2013:200
		ʔlupdep	thin (of object)	Washo Archive
		ʔudaw	tall (of object)	Washo Archive
	2	i:yel	big	Washo Archive
	3	hamham	light (in weight)	Washo Archive
		šɨšɨš	heavy	Washo Archive
Human propensity	1	bišapuʔ	hungry	Washo Archive
		gumbiʔis	proud	Washo Archive
		gumyoʔl	tired	Washo Archive
		k’iwɨl	sharp-thinking	Washo Archive
		Lokaš	afraid	Washo Archive
		meleyɨk	inebriated	Bochnak et al. 2011:5
		melot’ik	thirsty	Washo Archive
		šašɨw	afraid	Washo Archive
		t’e:su	jealous	Washo Archive
		yaha	sick/hurt	Washo Archive
		yomuŋ	full (from eating)	
		yumɨl	full (from eating)	
	2	gumsut’ɨm	brave	Washo Archive
		musiw	generous	Washo Archive
		tamugayʔl	bored	Washo Archive
Value	1	ʔaŋaw	good	Washo Archive
	2	mu:ʔaŋ	tasty	Washo Archive
		ʔnu:š	poor condition	
		ʔumbiʔic’	expensive	Washo Archive
Physical property	1	golgoš	short and fat	Washo Archive
		ibik’	ripe	Washo Archive
		ihuk’	dry	Washo Archive
		k’eše	alive	Washo Archive
		metuʔ	cold	Washo Archive
		mi:p’ɨl	full	Washo Archive
		mosot	wet	Washo Archive
		muc’uc’u	sweet	Washo Archive
		wɨhl	cold	Washo Archive
		yak’aš	warm	Washo Archive
		yasaŋ	hot	Washo Archive
		ʔyaʔyaŋ	naked	Washo Archive
	2	guc’u	torn	
		gumbeyéc’ɨk	closed	Washo Archive
		kakt	quiet	Washo Archive
		Loyaw	dark	
		nuʔuš	stinky	
		wkuliʔ	solid	Tribal dictionary
		yac’im	smoky	
	3	ba:bab	spotted	Washo Archive
		hu:hu	striped	Washo Archive
		k’awk’aw	closed	Jacobsen 1980:94
		k’unk’un	bent	Washo Archive
		kaykay	tall	Washo Archive
		kuškuš	short and fat	Washo Archive
		lotlot	soft	Washo Archive
		mukmuk	chubby	Washo Archive
		naynay	muddy	Washo Archive
		p’ep’el	bitter	
		p’ɨp’ɨ	thin	Tribal dictionary
		šapšap	fuzzy	Washo Archive
		ši:šip	straight	Jacobsen 1980:94
		sɨnsɨn	thin	Washo Archive
		siwsiw	smooth	Jacobsen 1980:90
		t’et’ep	fat	Washo Archive
		t’ɨnt’ɨn	rough	Washo Archive
		witwit	stiff	Washo Archive

Dixon (1982:16) lists the following categories of property concept: age, color, dimension, human propensity, physical property, speed, and value. In the dataset below, the categories with the most tokens fall in the categories physical property and human propensity. Note that there are no tokens from the speed category. This is because speed descriptors are only attested adverbially—as bound suffixes on motion predicates—as in the following examples with the affix *-ac’iw* ‘fast’ (cp., e.g., *-ayšay* ‘slowly’; see further discussion of ‘tempo’ suffixes in Jacobsen 1980:81).

The table shows that it is difficult to make predictions about which morphological class a given property concept should fall in based on Dixonian class. Consider for example the category age, whose antonym members—old and young—fall into different classes. The only fully predictable class seems to come from the category color, whose members are solely of Class 3. Class 1 has 30 total items, Class 2 has 15, and Class 3 has 35.

## Footnotes

1 We must emphasize that the term *property concept* as we use it here is purely descriptive and has no formal status in the discussion that follows. It is a term defined inductively as (i) an adjective or (ii) the primitive lexeme responsible for introducing the descriptive content of an adjective in a translationally equivalent sentence in English. See Francez and Koontz-Garboden (2017:21–23) for discussion.

2 We follow Leipzig glossing abbreviations for the Wá⋅šiw data in addition to the following: anim animate, attr attributive, inch inchoative, ind independent mood, opt optative, prosp prospective aspect, r reduplication, ss same subject (switch reference), stat static. A prefixed number represents intransitive subject agreement in verbs and possessive agreement in nouns. We use the standardized orthography for Wá⋅šiw adopted in Jacobsen 1964, which follows the IPA with the following exceptions in our examples: . Superscripted vowels (e.g., ^e^, ^E^) indicate vowel “coloring” (Jacobsen 1964:285–287). Stress is indicated with an acute accent. Examples labeled with ‘Washo Archive’ are taken from the corpus of examples available online at washo.uchicago.edu. Data without a reference come from Hanink’s fieldwork in Wá⋅šiw communities in California and Nevada between 2015–2024. The language situation is discussed in more detail in Sect. 3.1.

3 We accept that this likely means that portions and states are notational variants of one another, an issue which is irrelevant to our considerations, and in any event beyond the scope of this paper. See Bochnak et al. 2020 for further details and discussion.

4 See Stassen 2009 and Myler 2014, 2016 for overviews of possessive predication more generally.

5 After Lasersohn (1995), both states (of type *v*) and ordinary individuals (of type *e*) belong to the domain of individuals, meaning that both properties of individuals and properties of states can saturate the first argument of *have* in (15).

6 The Washo Archive is part of the larger Washo Documentation Project, which was founded in 2006 by Dr. Alan Yu at the University of Chicago. More information can be found at washo.uchicago.edu.

7 Wá⋅šiw lacks adjectives; when property concepts modify nouns they are nominalized from their verbal source and appear in apposition to the noun they modify (see Bochnak 2013).

8 We note that property concepts in this language have not hitherto been characterized in this way. While previous works have engaged with property concepts in Wá⋅šiw vis-à-vis comparison and gradability (Bochnak 2013, 2015) and the stage/individual-level distinction in predication (Bochnak et al. 2011), these works leave aside the morphological shape of property concepts (with some limited discussion of Class 3 morphology in Bochnak 2013:Ch. 5).

9 Because Jacobsen (1964:555) calls this suffix the ‘attributive-agentive’, this suffix is often glossed as attr ‘attributive’ in other work on Wá⋅šiw. We avoid this term in order to clearly distinguish this affix from the prefix *ʔil-*, which is also glossed as attr ‘attributive’. See footnote 10 and Sect. 5.2 for further discussion.

10 Jacobsen (1980:86) labels the prefix *ʔil-* simply as ‘p_r_’, signifying that it is a prefix that triggers reduplication (see Sect. 5.2). Bochnak (2013) glosses it as attr ‘attributive’; we follow that convention here.

11 It is worth noting, as pointed out by a reviewer, that our discussion, like Menon and Pancheva’s, is at least superficially tied up with the view that roots have meaning, independent of the syntactic context in which they are found—a view that has been challenged (see, e.g., Arad 2003; Acquaviva and Panagiotidis 2014; Harley 2014 for a discussion). Indeed, the Menon and Pancheva hypothesis is certainly about roots having (a particular kind) of meaning, as is our response in this paper. Having said that, it seems to us that this might be a question of expository convenience, in the sense that, when it comes to the details, we are really investigating roots in syntactic context (which can always be given their meaning postsyntactically via rules of allosemy, as in Harley 2014). Nevertheless, we do indeed observe correlations between behaviors *across* syntactic contexts, which we flesh out via different kinds of meanings for roots. Ultimately, because the point of our discussion is to engage with Menon and Pancheva’s hypothesis, we believe it is best to take it on its own terms. Whether our results, and indeed their hypothesis, could be stated in a fashion more consistent with the idea that “roots don’t have meaning” is a question that we believe would ultimately be one of notation, rather than empirical substance, so we do not explore it any further here.

12 Analyses of the positive form must account for the fact that that many property concept words give rise to truth conditions that are *vague*—they are sensitive to the context (see, e.g., Kennedy 2019 for overview and discussion). For instance, in degree-based approaches, vagueness is introduced by composition with (silent) pos (von Stechow 1984), which establishes a relative ordering above a contextual standard. In the present approach, vagueness is captured through contextual restriction of the existential quantifier (over Davidsonian states) as in Francez and Koontz-Garboden 2017.

13 Myler (2016) develops a theory of possessive predication in which possessive meaning comes not from the possessive morphosyntax itself, but rather from arguments. One aspect of the motivation for this is what he calls the ‘too many meanings problem’, whereby possessive morphosyntax does not seem to be semantically uniform. It remains to be seen quite how the facts discussed here, and their analytical consequences, would align with the kind of theory he articulates.

14 Within a larger discussion of bipartite verbs, Jacobsen (1980) analyzes only property concepts in Class 3, and treats *ʔil-…-iʔ* as an inseparable circumfix. In a more recent account, Bochnak (2013) explicitly treats both components of this morphology as semantically vacuous. See Sect. 5 for further discussion.

15 We assume, as is standard in Davidsonian semantics, that the stative argument that remains after predication undergoes default existential binding.

16 We note that Wá⋅šiw has been argued to be degreeless (Bochnak 2013, 2015). We have not discussed degrees above as part of our ontology; we assume, following Wellwood (2015, 2019) and others, that these can exist alongside an ontology of Davidsonian states like the one we have assumed here, in particular, as measures on states (Wellwood 2019:Ch. 8) (see Hanink and Koontz-Garboden 2021 for discussion). We follow Bochnak et al. (2020) in assuming that degrees are compositionally introduced by functional vocabulary, and that Wá⋅šiw is indeed degreeless in that it lacks functional vocabulary introducing degrees.

17 See Bochnak 2013:223–224 for evidence that these constructions entail the result state expressed by the property concept final.

18 Wá⋅šiw is a head-final language, as reflected in this schematic. We abstract away from the precise mechanism by which bipartite verbs are formed morphologically. While we must leave this question to future research, we note similarities between resultative bipartite verbs and resultative V-V compounds in, e.g., Mandarin (Lin 2004; Liu 2021; see also Hopperdietzel 2021 for a discussion).

19 A reviewer raises the important question of what we mean by ‘unattested’. What we mean by this term is that there are no such examples in the data sources available to us (existing work, recorded narratives, and fieldwork notes). We however cannot be entirely sure that such examples are impossible. Relatedly, the reviewer raises the question whether there might be some other semantic property that holds of Class 2 roots that makes it so that they fail to appear in bipartite verb constructions. For example, as Wechsler (2005) shows, the so-called ECM variety of resultative in English has the property that the secondary predicate must have a closed scale. Could it be that there are no closed scale roots in Class 2, and that, rather than their quality denotation, is what prevents them forming resultatives? Although definitively ruling out this hypothesis would require detailed work on the scalar structure of Class 2 roots, which is not possible both given the state of the language and the scope of this paper, it is reassuring that English translations of some of the Class 2 roots given in the Appendix are closed scale and prototypical examples of resultative secondary predicates in the English literature on the construction, e.g., *wkuliʔ* ‘solid’ and *gumbeyéc’ɨk* ‘closed’. That said, we agree with the reviewer’s broader point, which we take to be that at least in English, there are different kinds of resultatives, and they have different kinds of constraints, which are still being disentangled in the literature (see Beavers 2012 for an overview and discussion). It would therefore be hubristic to suggest that without a great deal of detailed work of the kind that has been conducted on English resultatives we can definitively rule out any alternative explanation for the Class 2 gap. That the gap does independently fit with our explanation of the morphology tied to property concept words in the language, however, we believe seems unlikely to be accidental.

20 As we have noted above, however, there are many analyses of resultative constructions, and many conceivable analyses of bipartite verb constructions that one might develop rooted in them. Could it be that, by adopting a different analysis of bipartite verb constructions, we could make it that Class 2 roots are predicted to appear in them (contrary to empirical observation)? While we are not prepared to say what is analytically impossible, the contrast in meaning that has been motivated for Class 2 versus Class 3 roots makes it either that i) type theoretically, they would not appear in the construction at all, as on the analysis developed above, or ii) they would be predicted not to appear in the construction because they would have an odd meaning. To see an analysis giving rise to this second type of ill-formedness suppose, for example, that rather than treating Class 3 roots like ‘straight’ in a Davidsonian fashion, we gave them denotations as ordinary predicates of individuals, as in (50a), a meaning for v_become_ more along the lines of the one in Dowty (1979) without an event semantics, as in (50b).

In keeping with the non-Davidsonian theory, Class 2 roots on such a theory could be treated as predicates of portions, as in Francez and Koontz-Garboden (2017), which, although making them predicates of a different type of ontological object, makes no substantive difference (Sect. 2.1). On such a theory, *i:yel* denotes a set of portions of bigness:

Now there is no type-theoretic mismatch in composing with v_become_. Instead, however, an odd meaning is generated for Class 2 roots in such a context. On such a theory, when a Class 3 root composes with v_become_, it creates a set of individuals that come to be in the set denoted by the Class 3 root, as in (50a) for ‘straight’. By contrast, if a Class 2 root composes with v_become_, the meaning yielded is a nonsensical one that we believe no speaker would ever need to talk about given the nature of our world. For example, with Class 2 (49) the meaning yielded would be as in (50b), i.e., the set of individuals that come to be bigness. Supposing one had a theory along these lines, we believe that the absence of Class 2 roots in this construction is still motivated by, and argues for, the quality-type meaning we have assigned to these roots. Rather than being unattested for type-theoretical reasons, however, on this theory they are unattested because they give rise to meanings that are never used in describing the world in which we live.

21 Jacobsen (1980:86) labels the prefix *ʔil-* as ‘p_r_’, signifying that it is a prefix that triggers reduplication (see fn. 10). The construction under discussion is the *single* environment in the language where *ʔil-* is ever found, thus excluding the possibility of comparison with independent data.

22 More recently, Yu (2012) has argued that the reduplication of the type in (51a) and (52a), alongside a few other contexts, is *full* CV(C) reduplication (e.g., *p’il* → *p’ilp’il* ‘blue’; *kay* → *kaykay* ‘tall’). In the full reduplication account, reduplication is always obligatory with property concept roots, but a second process of truncation follows in bipartite-verb finals (e.g., *ši:šip* → *i:šip* ‘straight’) that is absent with *ʔil-*prefixation. Yu (2012) shows that the majority of dependent forms in Wá⋅šiw are vowel-initial, and proposes that truncation of the initial consonant of property concept roots in bipartite verbs such as (53) “might be triggered by structural analogy to the predominance of vowel-initial dependent stems”, i.e., nonproperty concept finals in bipartite verbs such as those in (53) (from Jacobsen 1980):

As Yu (2012) discusses, it is dubitable that full reduplication is synchronically active in the language, most notably because the purported unreduplicated CV(C) roots are not attested in isolation anywhere in the language. Additionally, partial reduplication elsewhere in the language indicates plurality in the nominal domain, and pluractionality in the verbal domain (Jacobsen 1964; Yu 2005, 2012). As discussed by Yu (2012), it is more difficult to find a common semantic thread unifying cases of full reduplication (on the view that it exists). This pattern also appears in some (animal) names (*c’élc’el* ‘squirrel’), sound-related verbs (*wákwag* ‘to bark’), and semelfactives (*tukc’ímc’im* ‘to blink’); Yu suggests it may serve an iconic or onomatopoeic function. Crucially however, the semantic reflex typically associated with *partial* reduplication *is* attested in the finals of bipartite resultative constructions, for instance in (53), which involves repeated scratching actions:

Taking these points together, we do not take full reduplication (if it exists) to be active synchronically in the formation of property concept roots. On a related note, it is for these reasons that we do not think it prudent to ascribe our proposed type-shift, discussed below, to reduplication itself.

23 Stated more pedantically, ^▽^ is a function f taking a function g from individuals to a function i from states to truth values, to a function h from states s to 1 iff i(s)=1 and 0 otherwise.

24 A reviewer raises a concern about the use of a type-shifting operation despite assigning Class 3 roots a particular meaning. The type-shift proposed here is however well-motivated for the following reasons. Firstly, it has an overt morphophonological realization (*ʔil-*). Secondly, it is part of a combinatorial operation in that its appearance is tied to the appearance of the *-iʔ* affix, making its application predictable.

25 We take the difference between Class 1 and Class 3 roots to be a case of listedness, as the requirement for *ʔil-* only with the latter appears to be arbitrary, derived from the requirement that these forms may only appear as dependent forms (i.e., take a prefix). A reviewer points out that it might seem unusual for a listed subcase to constitute the majority, but offers a similar case from German: In German, the plural suffix *-en* is the most common, but also requires a list in that it is not as productive as plural *-s* (see Pinker 1999:Ch. 8).

26 This strategy can therefore be construed as a case of what Stassen (2009) terms ‘flexional’ predicativization. It is worth noting that this means that, as a reviewer notes, verbalizing predicativization exists independently of incorporation into HAVE-type verbs, a phenomenon that Myler (2016:345, fn.1) conjectured might be attested, but did not have any evidence for.

27 Wá⋅šiw does have a lexical verb *saʔ* with the broad meaning of ‘have’, ‘keep’, or ‘store’, used to express temporary states of possession.

The precise distinction between possessive *-iʔ* and *saʔ* is not entirely clear at present. Similar verbs are attested in Hiaki (Jelinek 1998) and Quechua (Myler 2014:Chap. 3). Notably, in these languages, possessive predication is also the result of functional possessive morphology rather than a lexical verb.

28 A similar set of affairs is found across Eskimo-Aleut, e.g., cognate *-qar* in Kalaallisut (Sadock 2003).

29 In other words, it is a bound morpheme that requires its complement to be a prefix. This could be the result of obligatory head movement as in the standard analysis (Baker 1988); Yuan (2018) argues that this is a postsyntactic requirement in Inuktitut. We remain agnostic on this issue.

30 Jelinek (1998) demonstrates related facts for Hiaki: note the accusative case-marking on the modifier in (65):

31 In the case of Inuktitut *-qaq*, Johns (2007) suggests an incorporation analysis of *have*, according to which *have* itself is a prepositional element that incorporates into a v_*be*_ head (Freeze 1992; Kayne 1993; Harley 1998), cf. Beavers et al. 2008; Myler 2014, 2018); Harley (2002) adopts a similar proposal for Hiaki -∅. We do not pursue this option here, but believe that such an analysis is compatible with our central claim.

32 A reviewer points out that it is unusual that the meaning of *ʔisaʔ* here is not treated as relational. How (or whether) relationality manifests in the language is a question we must leave to future research.

33 A parallel can be drawn here to notionally possessive *-ed* constructions in English such as *warm-hearted* or *brown-eyed* (Pesetsky 1995; Nevins and Myler 2014); notably, Nevins and Myler (2014) argue that the morpheme *-ed* is a root-categorizing adjectivizer (though Myler (2016:370) provides examples such as *a big-bumpered car* showing that it may also attach higher).

34 While Jacobsen (1964) classifies *-ašaʔ* as a tense marker, see Bochnak (2023) for arguments that it encodes prospective aspect.

35 Neither Hiaki nor Inuktitut allow their possessive light verbs to form property concepts; Trueman et al. (2019) consider this possibility for Hiaki but ultimately rule it out. This difference with Wá⋅šiw is likely attributable to the fact that both Hiaki and Inuktitut have dedicated adjectival categories, while Wá⋅šiw does not.
